# Piezo1, a novel therapeutic target to treat pulmonary arterial hypertension

**DOI:** 10.3389/fphys.2023.1084921

**Published:** 2023-01-26

**Authors:** Qifeng Yang, Xuanyi Li, Yue Xing, Yuqin Chen

**Affiliations:** State Key Laboratory of Respiratory Disease, National Center for Respiratory Medicine, National Clinical Research Center for Respiratory Disease, Guangdong Key Laboratory of Vascular Disease, Guangdong-Hong Kong-Macao Joint Laboratory of Respiratory Infectious Disease, Guangzhou Institute of Respiratory Health, the First Affiliated Hospital of Guangzhou Medical University, Guangzhou, China

**Keywords:** pulmonary arterial hypertension, Piezo1, calcium channel blocker, therapeutic targets, transient receptor potential channel (TRP channel)

## Introduction

Pulmonary arterial hypertension (PAH) is a life-threatening disorder characterized by Abrelevated mean pulmonary arterial pressure (mPAP >20 mmHg) as a consequence of enhanced pulmonary vascular resistance (PVR) (Simonneau et al., 2019). Pulmonary artery vasoconstriction and vascular remodeling greatly contribute to a sustained elevation of PVR and pulmonary arterial pressure (PAP) in patients with PAH. Abnormal upregulation of cytoplasmic Ca2+ in pulmonary artery smooth muscle cells plays a central role. However, calcium channel blocker (CCB) is only effective in 10% of patients with a positive acute vascular response and it is rapidly becoming resistant to treatment (Galie et al., 2015). The main pharmacological effect of CCB is the inhibition of L-type voltage-dependent calcium channels (VDCC), thus inhibiting receptor-operated Ca2+ channels (ROCC) which mainly regulates Ca2+ influx and consequently blocks the process of vasoconstriction (Ng and Gurney, 2001).

It is known that the main components of store-operated calcium channel (SOCC) are the members of the transient receptor potential channel (TRPC) family (Somlyo and Somlyo, Nature, 1994, 372, 231–236; Birnbaumer et al., Proc Natl Acad Sci United States, 1996, 93, 15,195–15202; Zhu et al., Cell, 1996, 85, 661–671; Zitt et al., Neuron, 1996, 16, 1,189–1,196) and pulmonary artery smooth muscle cell (PASMC) mainly expressed TRPC1, TRPC2, TRPC4, TRPC5, and TRPC6 (Golovina et al., Am J Physiol Heart Circ Physiol, 2001, 280, H746–755) **.** A mountain of studies (Golovina et al., Am J Physiol Heart Circ Physiol, 2001, 280, H746–755; Sweeney et al., Am J Physiol Lung Cell Mol Physiol, 2002, 283, L144–155; Fantozzi et al., Am J Physiol Lung Cell Mol Physiol, 2003, 285, L1233–1,245) has suggested that transient receptor potential channel played an important role in the development of PH, but few of them can be used as an effective therapeutic target**.** We find it difficult to target TRPC as a treatment target because of its wide implication. Du et al. (Du et al., FASEB J, 2014, 28, 4677–4685) described that TRPC1 can act as a component that senses shear stress.

It is generally recognized that shear stress, one of the most significant intravascular mechanics, plays a significant role in the contraction and remodeling of the vasculature. Piezo1 is a mechanosensitive, non-selective cationic ion channel protein. In specific, the Piezo channels are activated by shear stress in local blood flow and by cell membrane stretch (Douguet et al., 2019). We, therefore, suggest that Piezo1 is the initiating factor in the disturbance of Ca^2+^ homeostasis in PASMC in some types of PAH, for example, chronic thromboembolic pulmonary hypertension (CTEPH) and congenital heart disease-associated PAH**.** The primary roles of Piezo1 in vascular mechanical transduction have been identified as sensing blood flow shear stress and fostering vascular development (Li et al., 2014)**.** Piezo1 is localized at the subcellular organelles, including the endoplasmic reticulum (ER)/sarcoplasmic reticulum (SR), nucleus, and mitochondria; as well as the plasma membrane. Activation of Piezo1 increased intracellular free calcium concentration in a SOCE-independent manner (Liao et al., 2021) and over-expression of Piezo1 improved cell migratory velocity, showing Piezo1 involvement in cell motility (Maneshi et al., 2018). Additionally, it has been shown that effective knockdown of Piezo1 attenuated the FBS-induced proliferation of human PASMC (Maneshi et al., 2018) and inhibited the FBS-induced proliferation of human PASMC (Liao et al., 2021). Also, previous study (Chen et al., 2022)have shown that Piezo1 is required for pulmonary artery smooth muscle cell proliferation. Expression of Piezo1 is increased in the pulmonary artery endothelial cells of patients with idiopathic pulmonary artery hypertension and experimental PH (Wang et al., 2021) . In summary, Piezo1 expression is increased in both smooth muscle cells and endothelial cells of patients with PH and plays a pathological role.

Recently, some molecules have been found to inhibit Piezo1, some of which are derived from herbal ingredients, creating opportunities for the clinical application of Piezo1 as a therapeutic target. Salvia miltiorrhiza produces salvianolic acid B (SalB), a significant bioactive molecule that is water soluble**.** SalB shows a preference for Piezo1 channels and reduces the current caused by mechanical stimulation and Yoda1 stimulation, suggesting that it may have an impact on the vascular mechanical transduction in Piezo1 channels (Pan et al., 2022)**.** A possible mechanism for the inhibition of Piezo1 by SalB might be that it competitively inhibits the action of Yoda1^18^. A specific Piezo1 blocker is the peptide GsMTx4, which was extracted from the venom of the spider Grammostola spatulata (Suchyna et al., 2000). According to biophysical research, Piezo channels may be able to directly detect mechanical force when a lipid membrane is perturbed (Cox et al., 2016)**.** Amphipathic medicines, such as Aβ-peptides, modify membrane mechanics by changing the structure of the membrane (Williams and Serpell, 2011). Mohammad M. Maneshi and his colleagues (Maneshi et al., 2018) proved that enantiomeric Aβ peptides inhibit the fluid shear stress response of Piezo1, which inhibits Piezo1 from another possible mechanism by altering membrane structure and mechanics. The Chinese herb Bolbostemma paniculatum (Maxim) Franquet (Cucurbitaceae), often known as “Tu Bei Mu,” has a triterpenoid saponin called tubeimoside I (TBMS I) (Tang et al., 2015), which stands out as an efficient inhibitor of the Yoda1-response with selectivity for the Piezo1 channel (Liu et al., 2020) Figure 1. Based on the above studies, Piezo1 is promising as a target for the treatment of PAH. Piezo1 may act as an adaptive compensator in the initial stages of PAH, but Piezo1 acts as a pressure-sensing sensor, and changes in intravascular shear stress activate Piezo1, thereby disrupting calcium homeostasis. Therefore, Piezo1 may be a potential treatment target for PAH. Whereas further experiments are needed to confirm at which stage of PAH the intervention will have a positive effect.

**FIGURE 1:**
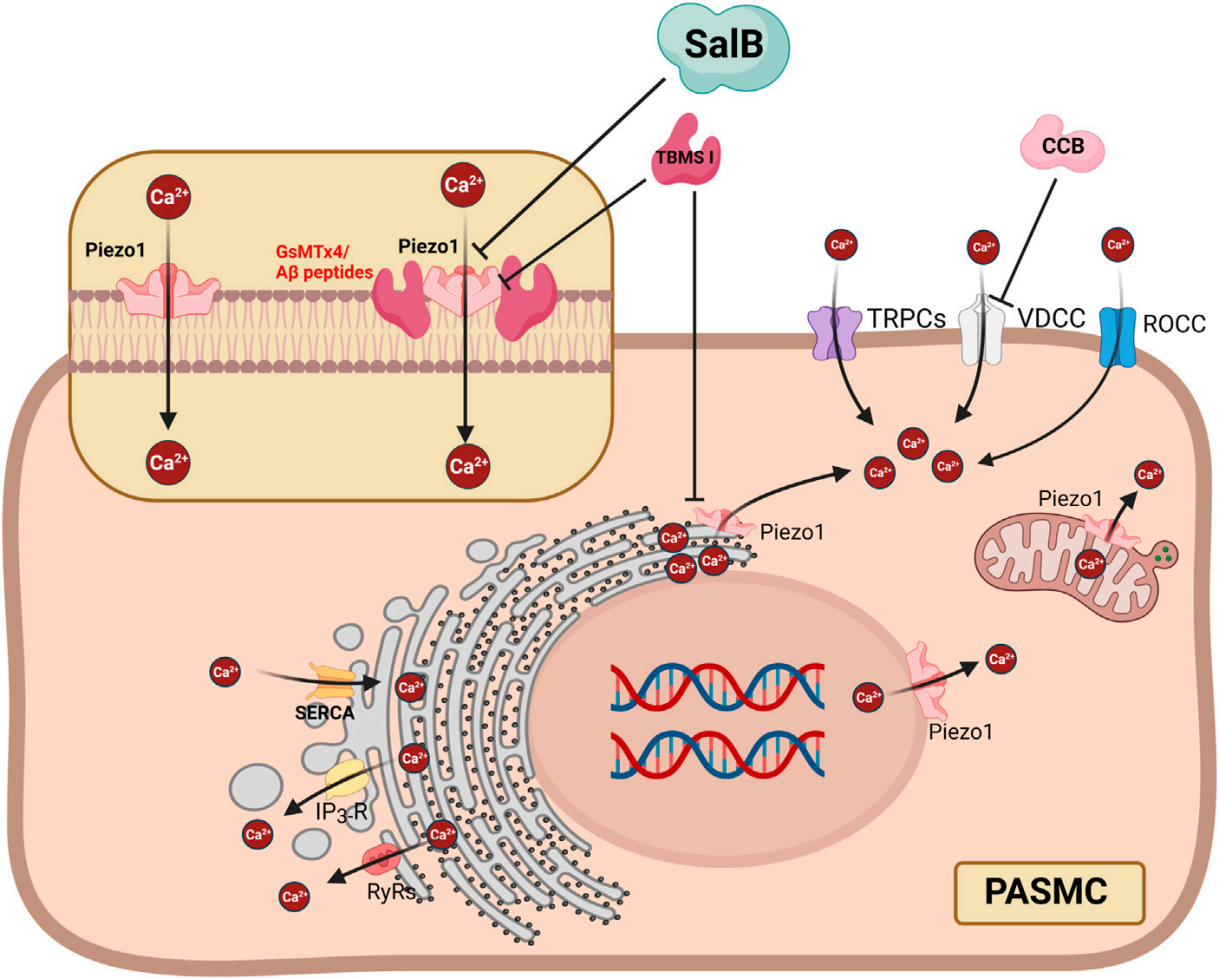
Proposed mechanisms showing that inhibition of Piezo1 in PASMC can suppress Ca^2+^ influx and release from subcellular organelles, as well as Ca^2+^ flow mediated by other Ca^2+^ channels. SalB can inhibit extracellular Ca^2+^ influx through the PM localized Piezo1 but not subcellular organelles. GsMTx4 and Aβ peptides inhibit Piezo1 by changing the structure of the membrane. TBSM I suppresses PM localized Piezo1 and subcellular organelles localized Piezo1. ER = Endoplasmic Reticulum; IP3R = inositol 1,4,5-trisphosphate receptors; PASMC = pulmonary artery smooth muscle cell; PM = plasma membrane; ROCC = receptor-operated Ca^2+^ channels; RyRs = ryanodine receptors; SalB = Salvianolic Acid B; SERCA = sarcoplasmic reticulum Ca [2+]-ATPase; SR = Sarcoplasmic Reticulum; VDCC = voltage-dependent calcium channels. The fiure is created with BioRender.com.

## References

[B1] Birnbaumer L. Zhu X. Jiang M. Boulay G. Peyton M. Vannier B. (1996). On the molecular basis and regulation of cellular capacitative calcium entry: Roles for trp proteins. Proc. Natl. Acad. Sci. U. S. A. 93, 15195–15202. 10.1073/pnas.93.26.15195 8986787 PMC26380

[B2] Chen J. Rodriguez M. Miao J. Liao J. Jain P. P. Zhao M. (2022). Mechanosensitive channel Piezo1 is required for pulmonary artery smooth muscle cell proliferation. Am. J. Physiol. Lung Cell Mol. Physiol. 322, L737–L760. 10.1152/ajplung.00447.2021 35318857 PMC9076422

[B3] Cox C. D. Bae C. Ziegler L. Hartley S. Nikolova-Krstevski V. Rohde P. R. (2016). Removal of the mechanoprotective influence of the cytoskeleton reveals PIEZO1 is gated by bilayer tension. Nat. Commun. 7, 10366. 10.1038/ncomms10366 26785635 PMC4735864

[B4] Douguet D. Patel A. Xu A. Vanhoutte P. M. Honore E. (2019). Piezo ion channels in cardiovascular mechanobiology. Trends Pharmacol. Sci. 40, 956–970. 10.1016/j.tips.2019.10.002 31704174

[B5] Du J. Ma X. Shen B. Huang Y. Birnbaumer L. Yao X. (2014). TRPV4, TRPC1, and TRPP2 assemble to form a flow-sensitive heteromeric channel. FASEB J. 28, 4677–4685. 10.1096/fj.14-251652 25114176 PMC4200325

[B6] Fantozzi I. Zhang S. Platoshyn O. Remillard C. V. Cowling R. T. Yuan J. X. J. (2003). Hypoxia increases AP-1 binding activity by enhancing capacitative Ca2+ entry in human pulmonary artery endothelial cells. Am. J. Physiol. Lung Cell Mol. Physiol. 285, L1233–L1245. 10.1152/ajplung.00445.2002 12909593

[B7] Galie N. Humbert M. Vachiery J. L. Gibbs S. Lang I. Torbicki A. (2015). 2015 ESC/ERS guidelines for the diagnosis and treatment of pulmonary hypertension: The joint task force for the diagnosis and treatment of pulmonary hypertension of the European society of cardiology (ESC) and the European respiratory society (ERS): Endorsed by: Association for European paediatric and congenital cardiology (AEPC), international society for heart and lung transplantation (ISHLT). Eur. Heart J. 37, 67–119. 10.1093/eurheartj/ehv317 26320113

[B8] Golovina V. A. PlatOshyn O. Bailey C. L. Wang J. LimsuwAn A. Sweeney M. (2001). Upregulated TRP and enhanced capacitative Ca(2+) entry in human pulmonary artery myocytes during proliferation. Am. J. Physiol. Heart Circ. Physiol. 280, H746–H755. 10.1152/ajpheart.2001.280.2.H746 11158974

[B9] Li J. Hou B. Tumova S. Muraki K. Bruns A. Ludlow M. J. (2014). Piezo1 integration of vascular architecture with physiological force. Nature 515, 279–282. 10.1038/nature13701 25119035 PMC4230887

[B10] Liao J. Lu W. Chen Y. Duan X. Zhang C. Luo X. (2021). Upregulation of Piezo1 (Piezo type mechanosensitive ion channel component 1) enhances the intracellular free calcium in pulmonary arterial smooth muscle cells from idiopathic pulmonary arterial hypertension patients. Hypertension 77, 1974–1989. 10.1161/HYPERTENSIONAHA.120.16629 33813851

[B11] Liu S. Pan X. Cheng W. Deng B. He Y. Zhang L. (2020). Tubeimoside I antagonizes yoda1-evoked Piezo1 channel activation. Front. Pharmacol. 11, 768. 10.3389/fphar.2020.00768 32523536 PMC7261832

[B12] Maneshi M. M. Ziegler L. Sachs F. Hua S. Z. Gottlieb P. A. (2018). Enantiomeric Aβ peptides inhibit the fluid shear stress response of PIEZO1. Sci. Rep. 8, 14267. 10.1038/s41598-018-32572-2 30250223 PMC6155315

[B13] Ng L. C. Gurney A. M. (2001). Store-operated channels mediate Ca(2+) influx and contraction in rat pulmonary artery. Circ. Res. 89, 923–929. 10.1161/hh2201.100315 11701620

[B14] Pan X. Wan R. Wang Y. Liu S. He Y. Deng B. (2022). Inhibition of chemically and mechanically activated Piezo1 channels as a mechanism for ameliorating atherosclerosis with salvianolic acid B. Br. J. Pharmacol. 179, 3778–3814. 10.1111/bph.15826 35194776

[B15] Simonneau G. Montani D. Celermajer D. S. Denton C. P. Gatzoulis M. A. Krowka M. (2019). Haemodynamic definitions and updated clinical classification of pulmonary hypertension. Eur. Respir. J. 53, 1801913. 10.1183/13993003.01913-2018 30545968 PMC6351336

[B16] Somlyo A. P. Somlyo A. V. (1994). Signal transduction and regulation in smooth muscle. Nature 372, 231–236. 10.1038/372231a0 7969467

[B17] Suchyna T. M. Johnson J. H. Hamer K. Leykam J. F. Gage D. A. Clemo H. F. (2000). Identification of a peptide toxin from Grammostola spatulata spider venom that blocks cation-selective stretch-activated channels. J. Gen. Physiol. 115, 583–598. 10.1085/jgp.115.5.583 10779316 PMC2217226

[B18] Sweeney M. Yu Y. Platoshyn O. Zhang S. McDaniel S. S. Yuan J. X. J. (2002). Inhibition of endogenous TRP1 decreases capacitative Ca2+ entry and attenuates pulmonary artery smooth muscle cell proliferation. Am. J. Physiol. Lung Cell Mol. Physiol. 283, L144–L155. 10.1152/ajplung.00412.2001 12060571

[B19] Tang Y. Li W. Cao J. Li W. Zhao Y. (2015). Bioassay-guided isolation and identification of cytotoxic compounds from Bolbostemma paniculatum. J. Ethnopharmacol. 169, 18–23. 10.1016/j.jep.2015.04.003 25882313

[B20] Wang Z. Chen J. Babicheva A. Jain P. P. Rodriguez M. Ayon R. J. (2021). Endothelial upregulation of mechanosensitive channel Piezo1 in pulmonary hypertension. Am. J. Physiol. Cell Physiol. 321, C1010–C1027. 10.1152/ajpcell.00147.2021 34669509 PMC8714987

[B21] Williams T. L. Serpell L. C. (2011). Membrane and surface interactions of Alzheimer's Aβ peptide--insights into the mechanism of cytotoxicity. FEBS J. 278, 3905–3917. 10.1111/j.1742-4658.2011.08228.x 21722314

[B22] Zhu X. Jiang M. Peyton M. Boulay G. HuRst R. StEfani E. (1996). trp, a novel mammalian gene family essential for agonist-activated capacitative Ca2+ entry. Cell 85, 661–671. 10.1016/s0092-8674(00)81233-7 8646775

[B23] Zitt C. Zobel A. Obukhov A. G. HarteneCk C. Kalkbrenner F. Luckhoff A. (1996). Cloning and functional expression of a human Ca2+-permeable cation channel activated by calcium store depletion. Neuron 16, 1189–1196. 10.1016/s0896-6273(00)80145-2 8663995

